# Prospective payment system and racial/ethnic disparities: a national retrospective observational study in anaemia complication among end-stage renal disease patients in the US

**DOI:** 10.1186/s12882-020-02081-4

**Published:** 2020-10-06

**Authors:** Nga TQ. Nguyen, Alexander P. Maxwell, Michael Donnelly, Ciaran O’Neill

**Affiliations:** Centre for Public Health, School of Medicine, Dentistry and Biomedical Sciences, Queen’s University Belfast, Belfast, UK

**Keywords:** Anaemia, end-stage renal disease, Racial/ethnic disparities, Prospective payment system

## Abstract

**Background:**

A series of policy changes in 2011 altered reimbursement arrangements and guidance on use of erythropoiesis-stimulating agents for end-stage renal disease (ESRD) patients with anaemia in the US. While the policy changes were principally directed at care delivered in an outpatient setting, these had the potential to affect inpatient care also. This study used HCUP-NIS data (2008–2016) to examine trends in recorded anaemia among ESRD hospitalizations and analyse disparities in inpatient outcomes among ethnic groups following policy changes.

**Methods:**

The International Classification of Diseases codes were used to identify ESRD admissions, recorded anaemia due to chronic kidney disease (CKD), and to generate an age-adjusted Deyo-Charlson comorbidity index. Linear splines with a knot placed at the time point of policy changes and multivariable logistic regression were used to examine the likelihood of recorded anaemia, adjusted for a range of socio-demographic and clinical covariates. Difference-in-difference analyses investigated the impact of policies on recorded anaemia. Other outcomes included hospital cost, mortality and place of discharge.

**Results:**

The percentage of inpatient episodes with recorded anaemia arising from CKD increased from 26.2% in 2008 to 50.0% in 2016. Anaemia was more likely to be recorded as a complication of ESRD among minority ethnic groups and Native American admissions, in particular, (OR 1.20, 95%CI 1.15–1.25) relative to White American admissions; and these disparities widened following changes to reimbursement. Minorities were less likely to die in hospital and to be discharged to another healthcare facility, and (with the exception of Black American admissions) they were more expensive to treat.

**Conclusions:**

Our findings provide evidence of an increase in recorded anaemia consistent with a shift of patients from outpatient to inpatient settings in the wake of changes to reimbursement enacted in 2011. In addition, the study highlights the existence of ethnic disparities that widened after the policy initiated reimbursement changes.

## Background

End-stage renal disease (ESRD) affected more than half a million people in the United States (US) in 2016 and is associated with a significant economic burden [1]. Anaemia is among the most commonly diagnosed modifiable complications of chronic kidney disease (CKD) and is more pronounced in later stages of the disease [2]. Anaemia is associated with higher risks for the development of left ventricular hypertrophy, heart failure [3], cognitive impairment [4], and poorer quality of life [5]; it is also an important predictor of mortality [6].

The pathogenesis of anaemia in CKD is multifactorial but the central problem is reduced erythropoietin production by the kidneys [2, 7]. While erythropoiesis-stimulating agents (ESAs) remain the first-line treatment for anaemia, serious concerns about their safety have been raised [8], and helped prompt policy changes related to their use in the US [9]. In 2011, a series of policy changes adopted in the US combined to change incentives around the use of ESAs. The Medicare ESRD prospective payment system (PPS) implemented in 2011 altered financial incentives by allowing providers to retain payments above Medicare’s reimbursement level while providing dialysis services. This reduced the incentive to provide treatments formerly reimbursed on a fee-for-service basis notably injectable medications such as ESAs [9]. In the same year, the US Food and Drug Administration (FDA) revised the prescribing advice for ESAs with guidance on a more conservative use of ESAs for renal anaemia [10]. Also in 2011 the ESRD Quality Incentive Payment was implemented [11]. This program aimed to raise care quality by imposing a penalty for poor management of dialysis patients including the overuse of ESAs [11]. These three policy changes altered the financial context in which anaemia was managed principally in an outpatient setting. All constrained the use of ESAs. Hereafter these changes are for convenience referred to as PPS. While there is some evidence that the policy changes resulted in shift in transfusions from an outpatient to an inpatient setting [12], their effect on care and outcomes in an inpatient setting more generally have not to our knowledge been examined.

Race/ethnicity adds a layer of complexity to the potential impact of these policy changes. Ethnicity has been shown to be predictive of anaemia [5] and while ethnic disparities have been well documented in the literature for Black and White Americans [13, 14], there is limited evidence on the comparative experience of other ethnic groups or what impact the changing policy context has had in respect of them. This may be due in part to the smaller numbers of specific groups such as Native Americans who might appear in survey data [5]) or a lack of life tables for these populations [15]. These groups also experience economic disadvantage [16] and with regard to Native Americans, have the poorest renal outcomes and highest age-adjusted prevalence of diagnosed diabetes among the various ethnic groups [17]. Therefore, an examination of their specific experience may be insightful. The aim of this study was to investigate the impact of PPS on the likelihood of an inpatient episode for ESRD being recorded with anaemia following the adoption of PPS as well as its effect on hospital costs, inpatient mortality and discharge destination. A secondary aim was to examine the impact of PPS on ethnic disparities with a particular focus on the experience of Native Americans.

## Methods

### Data sources

Data were obtained from the National (Nationwide) Inpatient Sample - Healthcare Cost and Utilization Project (HCUP-NIS), Agency for Healthcare Research and Quality (AHRQ) [18]. HCUP-NIS is the largest all-payer inpatient care database in the US representing an approximately 20% sample of discharges from all HCUP community hospitals, excluding rehabilitation and long-term acute care hospitals in the US. HCUP-NIS data contains over 7 million hospital admissions annually, which represents over 35 million hospitalizations every year in the US [18]. We pooled 9 years data (2008–2016) to increase sample size and permit analyses of policy changes.

The International Classification of Diseases 9th and 10th Revision (ICD9 and ICD10) codes were used to identify all admissions with a primary diagnosis of ESRD (ICD9 585.6, ICD10 N18.6) and a procedure/diagnosis code referring to any type of renal replacement therapy (RRT, comprising: haemodialysis, peritoneal dialysis and kidney transplantation). Episodes with a diagnosis code of acute kidney injury (ICD9 584.x, ICD10 N17.x) were excluded from study because they represented a distinct patient cohort [19]. From this cohort, episodes that involved anaemia due to CKD were identified using ICD codes. Additional file 1 (Table S1) provides details on ICD9 and ICD10 codes used. An Age-adjusted Deyo-Charlson Comorbidity Index (ACCI) was developed based on ICD codes for each patient’s episode of care [20]. We also determined iron deficiency status using ICD codes for inclusion in analyses.

### Data analyses

Sample characteristics were summarized using mean and standard deviation (SD) for continuous variables and proportions for categorical variables. Ethnicities were grouped into six categories: White, Black, Hispanic, Asian, Native American and Others based on data in HCUP-NIS.

To examine the impact of PPS on anaemia outcome, we stratified our sample by two periods: before PPS (2008 to 2010) and after PPS (2011 to 2016) [21]. Linear splines with one knot placed at 2011 and logistic regression models were used to estimate trends over time taking the timing of PPS into account. This was consistent with previous studies [12, 21] and an preliminary examination of our data (Fig. S1, Additional file). Models were adjusted for a range of socio-demographic (insurance type, gender, age at admission, median household income for patient’s ZIP Code and location -broadly in terms of rurality), and clinical variables (renal replacement therapies, iron deficiency, comorbidity score ACCI, hospital characteristics, proteinuria, diabetes with or without complications) (see Additional file 1 for more details).

Marginal effects were used to estimate the trend in recorded anaemia across ethnicities, gender, type of RRT, income quartiles and type of insurance over the 9-year period. A difference-in-difference analysis was conducted by using marginal effects to estimate the gap between White Americans and other races/ethnicities before and after the adoption of PPS.

To examine the potential for PPS’s effect to extend more deeply into inpatient care, we examined the likelihood of inpatient mortality and discharge to a healthcare facility among those admitted with a primary diagnosis of ESRD using multivariable logistic regression. Discharge destination included routine discharge and discharge to healthcare facilities (including long-term care facilities or care homes, short-term hospitals, home healthcare and other rehabilitation centers), excluding those who died at the hospital. Models were adjusted for socio-demographic and clinical variables. Charges were converted to costs using the ‘cost-to-charge’ ratio tool provided by AHRQ-HCUP. Hospital costs were then adjusted for inflation using the personal consumption expenditure health component price index based on its ability to capture information on expenditures by all payers [22]. Generalized linear models (GLM) were used to accommodate the continuous, positive and skewed nature of hospital cost data in the cost analysis. Akaike information criterion and Bayesian information criterion were used to assess the fit of the GLM model [23].

### Sensitivity analyses

In order to generate national estimates using HCUP-NIS data that span multiple years, we conducted a sensitivity analysis with all models weighted by trend weight for data years prior to 2012 and by the discharge-level weight for data years 2012 and later as recommended by the AHRQ [18].

## Results

### Characteristics of study population

Among 591,683 admissions with ESRD over a 9-year period, the proportion of episodes of care related to White, Native, Black, Asian, and Hispanic Americans were 39.8, 0.95, 36.6, 3.4, and 16.4% respectively (Table 1). The percentage of inpatient episodes with recorded anaemia arising from CKD increased from 26.2% in 2008 to 50.0% in 2016.

**Table 1:** Descriptive statistics of the pooled sample from 2008 to 2016 (*N* = 591,683)

	White American (*n* = 235,227)		Native American (*n* = 5621)		Black American (*n* = 216,784)		Asian American (*n* = 20,342)		Hispanic American (*n* = 97,220)	
	N	%	N	%	N	%	N	%	N	%
Female	108,233	46.0	3131	55.7	113,531	52.4	10,293	50.6	47,310	48.7
Age at admission (Mean SD)	64.2	15.9	58.0	14.7	57.0	15.7	64.8	15.7	57.5	16.4
Insurance type										
Medicare	192,021	81.6	4346	77.3	165,094	76.2	14,701	72.3	61,433	63.2
Medicaid	12,233	5.2	658	11.7	29,473	13.6	2824	13.9	20,835	21.4
Private insurance	26,433	11.2	400	7.1	17,696	8.2	2223	10.9	8208	8.4
Others	4540	1.9	217	3.9	4521	2.1	594	2.9	6744	6.9
Location										
Central counties	55,501	23.6	1201	21.4	102,810	47.4	11,910	58.6	53,108	54.6
Large metro	63,697	27.1	581	10.3	45,326	20.9	3938	19.4	13,844	14.2
Medium metro	48,347	20.6	968	17.2	32,097	14.8	3247	16.0	20,148	20.7
Small metro	24,072	10.2	690	12.3	15,787	7.3	619	3.0	4954	5.1
Micropolitan	26,629	11.3	1199	21.3	13,019	6.0	561	2.8	3724	3.8
Not metropolitan or Micropolitan	16,981	7.2	982	17.5	7745	3.6	67	0.3	1442	1.5
Median household income for patient’s ZIP Code^a^										
First quartile	63,311	26.9	3039	54.1	118,330	54.6	3338	16.4	43,100	44.3
Second quartile	65,208	27.7	1368	24.3	45,241	20.9	4065	20.0	23,166	23.8
Third quartile	59,311	25.2	846	15.1	33,093	15.3	5766	28.4	20,292	20.9
Fourth quartile	47,397	20.2	368	6.6	20,120	9.3	7173	35.3	10,662	11.0
Elective admission	21,180	9.0	495	8.8	12,859	5.9	1208	5.9	6664	6.9
ACCI (Mean SD)	6.4	2.5	5.7	2.2	5.6	2.4	6.4	2.5	5.6	2.4
RRT modality										
Transplantation	13,443	5.7	214	3.8	7379	3.4	1107	5.4	3878	4.0
Hemodialysis	208,911	88.8	5197	92.5	201,176	92.8	18,140	89.2	89,591	92.2
Peritoneal analysis	12,873	5.5	210	3.7	8229	3.8	1095	5.4	3751	3.9
Diabetes without complications										
Yes	59,358	25.2	1642	29.2	58,213	26.9	5792	28.5	28,333	29.1
Diabetes with complications										
Yes	64,978	27.6	2398	42.7	51,406	23.7	6839	33.6	33,745	34.7
Anaemia due to CKD										
Yes	88,791	37.8	2380	42.3	83,310	38.4	8730	42.9	40,428	41.6
Iron deficiency										
Yes	4331	1.8	93	1.7	4027	1.9	346	1.7	1741	1.8
Die during hospitalization										
Yes	5123	2.2	67	1.2	2459	1.1	351	1.7	1120	1.2
Discharge destination: Health care facility										
Yes	96,895	41.2	1460	26.0	69,638	32.1	6482	31.9	25,214	25.9
Hospital characteristics										
Private hospital	189,042	80.4	4596	81.8	168,488	77.7	16,140	79.3	74,132	76.3
Hospital in urban area	192,126	81.7	4262	75.8	181,126	83.6	17,970	88.3	85,945	88.4
Teaching hospital	109,764	46.7	2674	47.6	124,544	57.5	10,127	49.8	48,633	50.0

Note: ^a^Income quartiles presented in this table are the estimated median household income of residents in the patient’s ZIP Code. The quartiles are identified by values of 1 to 4 indicating the poorest (first quartile) to wealthiest populations (fourth quartile)

The mean age at admission ranged from 57.0 (±15.7) for Black American admissions to 64.8 (±15.7) for Asian American admissions. Medicare was the predominant type of insurance while only a small percentage had private insurance in each racial group, with the smallest number observed in Native American admissions (7.1%). A higher percentage of Native American admissions were from smaller sized communities compared to other ethnicities. More than half of Native American and Black American admissions lived in areas included in the lowest quartile of median income.

Regarding the modality of RRT, a large proportion of the sample received haemodialysis with the highest percentage observed among Black (92.8%) followed by Native American admissions (92.5%). Diabetes was most prevalent in admissions who were Native Americans with 42.7 and 29.2% of them having diabetes with and without complications, respectively. Approximately 42.3% of episodes among Native Americans and 37.8% of those among Whites with ESRD had anaemia recorded as a diagnosis.

### Disparities in recorded anaemia

Ethnicity remained a strong predictor of anaemia as a recorded complication after controlling for a range of demographic, socioeconomic and clinical factors (Table 2). In the fully adjusted model, compared to admissions who were White Americans, the odds of anaemia was highest for those who were Native Americans (OR 1.20, 95%CI 1.15–1.25), and Asians (OR 1.20, 95%CI 1.17–1.22), followed by Hispanics (OR 1.12, 95%CI 1.10–1.13) and Blacks (OR 1.04, 95%CI 1.03–1.05). Admissions who had iron deficiency, diabetes with complications, lower ACCI score, who were aged less than 50 years or more than 80 years had a higher risk of anaemia compared to other groups (Additional file 1, Table S2).

**Table 2:** Relative likelihood of inpatient CKD-related anaemia among ESRD admissions by ethnicity

	Model 1			Model 2			Model 3			Model 4		
	OR	95% CI		OR	95% CI		OR	95% CI		OR	95% CI	
White Americans		(Reference)			(Reference)			(Reference)			(Reference)	
Native Americans	1.35^***^	1.30	1.40	1.23^***^	1.18	1.28	1.29^***^	1.24	1.34	1.20^***^	1.15	1.25
Black Americans	1.04^***^	1.04	1.05	1.04^***^	1.03	1.05	1.04^***^	1.03	1.05	1.04^***^	1.03	1.05
Asian Americans	1.23^***^	1.21	1.26	1.20^***^	1.18	1.23	1.21^***^	1.19	1.24	1.20^***^	1.17	1.22
Hispanic Americans	1.17^***^	1.16	1.18	1.13^***^	1.12	1.15	1.13^***^	1.12	1.14	1.12^***^	1.10	1.13

Note: Model 1: unadjusted model logistic regression model. Model 2, 3, 4: linear splines with one knot placed at 2011 and logistic regression models. Model 2: adjusted for demographic and socioeconomic variables (insurance type, gender, age at admission, median household income for patient’s ZIP Code, location). Model 3: adjusted for clinical variables (renal replacement therapies, iron deficiency, proteinuria, age adjusted comorbidity score ACCI, hospital characteristics, diabetes with or without complications). Model 4: fully adjusted model for all demographic, socioeconomic and clinical variables.* *p* < 0.05, ** *p* < 0.01, *** *p* < 0.001. CI – Confidence intervals

**Fig. 1:**
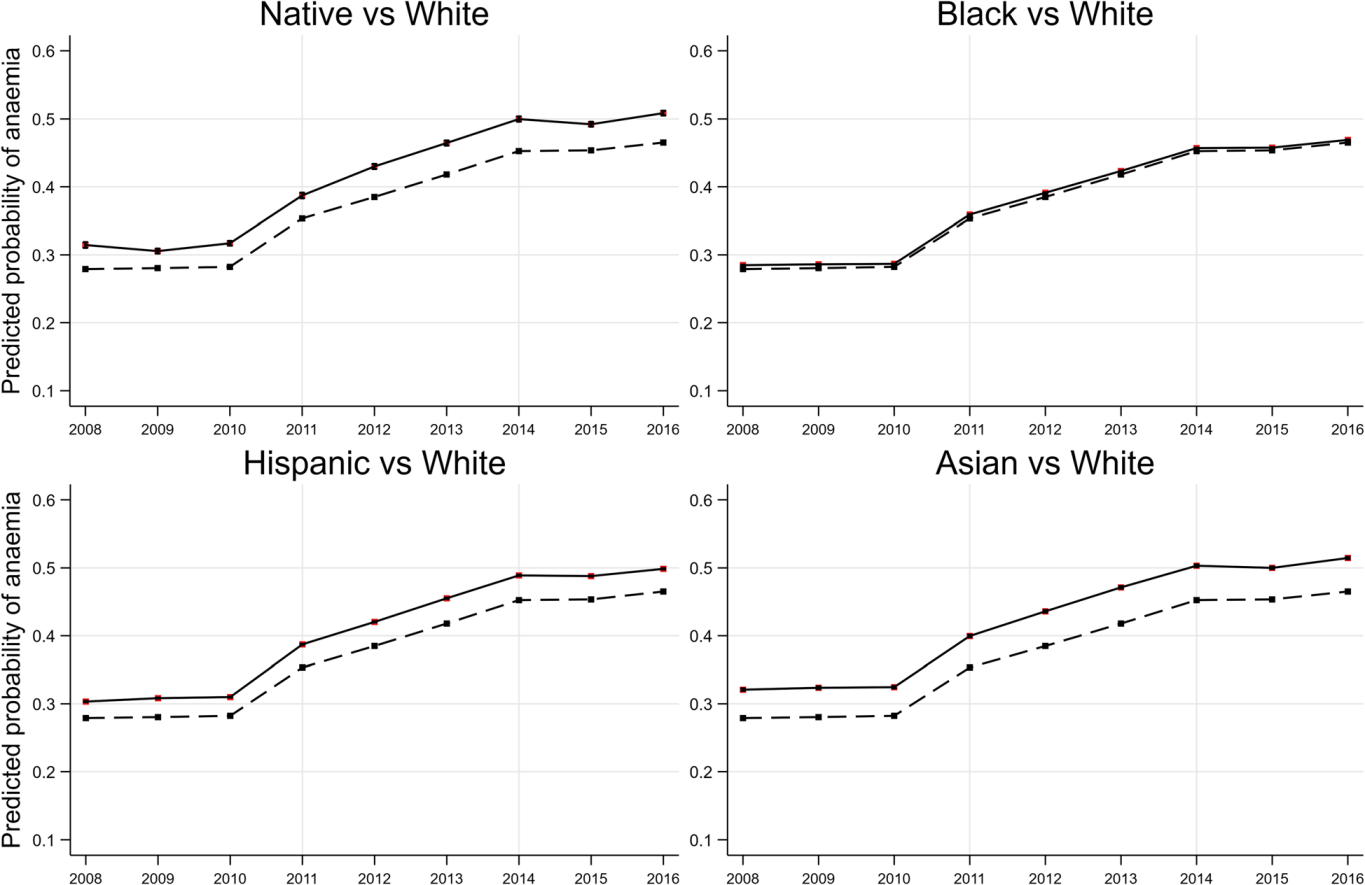
Predicted probability of anaemia from the fully adjusted model across ethnic groups compared to White admissions

Figures 1 depicts the likelihood of being diagnosed with anaemia over the 9-year period across ethnicity. The overall trend is consistent across all comparisons: all ethnicities have a higher likelihood of anaemia than White Americans admissions and there is a sharp increase in the likelihood of anaemia for all ethnicities in 2011 followed by a steady increment afterwards (Fig. 1). Comparisons across gender, type of RRT, income quartile and type of insurance groups are presented in Additional file 1 (Fig. S2). Those admissions whose RRT were via transplantation had a lower likelihood of anaemia compared to those on haemodialysis; those with private insurance or Medicare had the lowest predicted probability of anaemia followed by those on Medicaid and then other types of insurance.

### The impact of PPS on recorded anaemia

Linear splines with a knot placed at 2011 and logistic regression analysis were used to investigate the impact of PPS on anaemia outcome. Results from the fully adjusted regression model showed that the odds of recorded anaemia after PPS was 1.42 (95%CI: 1.40–1.44) compared to the preceding period. Difference in difference (DiD) analysis – looking at the experience of specific races/ethnicities over different periods – combined with marginal effect analysis revealed that the changes in the difference between racial groups before and after the introduction of PPS were significant, where the gap was most evident for admission who were Native Americans and Asian Americans (Table 3). For example, before PPS, the probability of recorded anaemia was 3.67% higher in Native American admissions relative to White American admissions. This difference increased in the following periods by 0.57% (95%CI: 0.42–0.72%, *p* < 0.001) to 4.25%.

**Table 3:** Impact of policy on racial disparities

	Before PPS	After PPS	Difference in difference	95%CI
White Americans	Reference			
Native Americans	3.67%	4.25%	0.57%***	0.42–0.72%
Black Americans	0.40%	0.48%	0.07%***	0.02–0.12%
Asian Americans	3.74%	4.33%	0.59%***	0.50–0.67%
Hispanic Americans	2.08%	2.44%	0.36%***	0.30–0.42%

Note: This table presents the marginal effect analysis results from Model 4 (Table 2) in form of incremental probability of having recorded anaemia of different ethnicities vs White Americans. *** *p* < 0.001

### Inpatient mortality, discharge destination, and hospital costs

In the wake of PPS, the odds of inpatient mortality for admissions with anaemia compared to admissions without anaemia increased; the odds ratio for admissions with anaemia dying in hospital rose from 0.61 (95%CI: 0.56–0.67) to 0.72 (95%CI: 0.68–0.76) between 2008 and 2010 and 2011–2016 (Table 4). The likelihood of discharge to another healthcare facility increased, and costs per inpatient episode fell (Table 4). Minorities including Native American admissions were less likely to die in hospital, less likely to be discharged to another healthcare facility and were more expensive to treat (with the exception of Black American admissions who had lower costs) (Table 5).

**Table 4:** Changes in key outcomes before and after PPS adoption

	Before PPS			After PPS		
	N (%)	*p*-value^Φ^	OR	N (%)	*p*-value^Φ^	OR
Inpatient mortality ^a^						
No anaemia ^α^	2594 (2.1)		Ref	3993 (1.68)		Ref
Anaemia ^β^	645 (1.29)	***	0.61*** (0.56–0.67)	2160 (1.2)	***	0.72*** (0.68–0.76)
Discharge to a healthcare facility ^b^						
No anaemia ^α^	41,606 (35.3)		Ref	83,812 (36.7)		Ref
Anaemia ^β^	16,150 (33.4)	***	0.93*** (0.91–0.95)	63,387 (36.5)	0.30	1.00 (1.00–1.02)
	Mean (SD)	*p*-value^¥^	Marginal effects	Mean (SD)	*p*-value^¥^	Marginal effects
Hospital cost (2016 USD) ^c^						
No anaemia	11,565 (14623)		Ref	10,859 (14454)		Ref
Anaemia	11,352 (13032)	**	768*** (654–882)	10,790 (12480)	0.11	683*** (615–752)

Note: ^a,b,c^ All models were adjusted for demographic and socioeconomic variables (insurance type, gender, age at admission, median household income for patient’s ZIP Code, location), clinical variables (renal replacement therapies, iron deficiency, proteinuria, age adjusted comorbidity score ACCI, hospital characteristics, diabetes with or without complications), and year of admission. ^a^ Logistic regression model; ^b^ Logistic regression model, restricted to admissions who were alive at discharge, ^c^ Generalized linear models, additionally adjusted for inpatient mortality. OR: odds ratio, 95%CI: 95% confidence interval. Marginal effect estimates are presented in form of incremental/decremented hospital cost. ^α^The number (percentage) of admissions who died in hospitals/were discharged to a healthcare facility and who did not have anaemia diagnosis, ^β^The number (percentage) of admissions who died in hospitals/were discharged to a healthcare facility and who had anaemia diagnosis. ^Φ^ Chi square test, ^¥^ Independent sample T-test. Ref: reference group. * *p* < 0.05, ** *p* < 0.01, *** *p* < 0.001

**Table 5:** Racial disparities in inpatient mortality, discharge destination, and hospital costs among ESRD admissions

	Inpatient mortality ^a^		Discharge to a health facility ^b^		Hospital cost (2016 USD) ^c^	
	OR	95%CI	OR	95%CI	Marginal effects	95%CI
White Americans	Ref		Ref		Ref	
Native Americans	0.78*	0.61–0.99	0.59***	0.55–0.63	364*	82 to 646
Black Americans	0.77***	0.73–0.81	0.89***	0.88–0.90	− 142***	− 212 to −72
Asian Americans	0.88*	0.79–0.99	0.60***	0.58–0.62	1394***	1221 to 1567
Hispanic Americans	0.81***	0.75–0.86	0.61***	0.60–0.63	20	−71 to 113

Note:: ^a,b,c^ All models were adjusted for demographic and socioeconomic variables (insurance type, gender, age at admission, median household income for patient’s ZIP Code, location), clinical variables (renal replacement therapies, iron deficiency, proteinuria, age adjusted comorbidity score ACCI, hospital characteristics, diabetes with or without complications. ^a,b,c^ Spline models with one knot placed at 2011 and ^a^ Logistic regression model; ^b^ Logistic regression model, restricted to admissions who were alive at discharge, ^c^ Generalized linear models, additionally adjusted for inpatient mortality. OR: odds ratio, 95%CI: 95% confidence interval. Marginal effect estimates are presented in form of incremental/decremented hospital cost of different ethnicities vs White Americans. Ref: reference group. * *p* < 0.05, ** *p* < 0.01, *** *p* < 0.001

### Sensitivity analyses

Results from sensitivity analyses in which HCUP weights are applied to the sample are presented in Additional file 1 (Table S3). It is evident that after being weighted using HCUP weights, ethnic disparities between admission who were Whites and other ethnicities remained significant with the highest ORs observed in Native American, followed by Asian, Hispanic and Black American admissions. The sensitivity analysis results were almost identical compared to the main results with regards to ethnic groups.

## Discussion

We found that anaemia as a recorded complication of ESRD in an inpatient setting increased significantly in the wake of the introduction of PPS in 2011 and has continued on an upward trend thereafter. The findings are consistent with those of Wetmore et al. (2016) in respect of transfusions in the sense that the structural break in 2011 may have been associated with a shift in the delivery of care for this patient group from an outpatient to an inpatient setting [12]. As can be seen in Table 4, while the fall in per patient costs has attenuated the increased financial burden associated with the management of anaemia in this setting this has been more than offset by the increased numbers being treated.

Ethnicity was a strong predictor of recorded anaemia due to CKD among ERSD inpatients. Other studies also found that Blacks [5, 24] and Hispanics [5] had significantly higher odds of anaemia compared to Whites. However, we found the disparity experienced by Native American admissions was greater than that of any other ethnic group. A review in 2011 found that anaemia was a public health problem among indigenous populations internationally [25]. While iron deficiency is the most common cause of anaemia in the US, iron deficiency prevalence has been reported to be 10-times higher among Native Americans [26]. Other studies found that Native Americans might be more likely to suffer anaemia as a result of delayed treatment, or poorer self-care [27]. As complications of ESRD are inter-related and due to the chronicity of the disease, whether Native Americans could have more complications other than anaemia (i.e. bone and mineral disorders, malnutrition, metabolic acidosis) requires further research.

While sharper disparities in terms of magnitude with respect to cost between ethnicities were observed (Table 5), the interpretation of these results require caution. Costs reflect length of stay and the procedures associated with an admission, and these entities may be influenced by disease severity and case complexity. Unfortunately, HCUP provided limited data with respect to severity/complexity though the observation that non-White admissions had a higher percentage of diabetes and diabetes with complications is consistent with greater complexity and higher costs (see Table 1). Moreover, we found that admissions with anaemia were less likely to die in the hospital compared to those without this condition. Given the data available we can only speculate as to what may explain this seemingly counter intuitive result. It is possible that those with a diagnosis of anaemia are more closely monitored/aggressively managed by staff. It is also possible that an element of selection bias results in a group of relatively healthy patients who would formerly have been adequately managed in ambulatory care now be transferred to inpatient care with more positive outcomes. This is an area that warrants further research.

The linear splines analysis highlighted the response among ESRD inpatients to policy changes (Fig. 1 and Table 3). There was an evident upward trend in the prevalence of recorded anaemia after the 2011. Thamer et al. (2014) also reported that major declines in ESAs doses were observed in most large dialysis providers in the US due to the confluence of financial incentives bundling payment for these medications in the first year after PPS [28]. Although Turenne et al. (2015) found no meaningful differences by race/ethnicity regarding the rates of change of management practices or laboratory measures in anaemia after PPS, this study was conducted in a comparatively short window (17 months) during which effects might not be evident [21]. Moreover, during the study period (2008–2016), the standardised incidence of ESRD among White Americans stabilized while it decreased slightly for Black and Native Americans (though remained 2.9 and 1.2 times higher compared to Whites) [1]. However, given the welcoming decrease in incidence, the increased likelihood of anaemia found among Native Americans after policy changes warrants further research.

Our study has shown that the adoption of policies intended to manage the use of ESAs might have unintended consequences such as changes in resource use and where care is delivered. Our study demonstrates an increase in episodes of care for such patients in an inpatient setting and a widening of ethnic disparities in such care. The reduction in cost and increase in mortality suggest changes in intensity of care and outcomes experienced by the patient cohort being admitted to hospital. Studying such changes is important in ascertaining the affect across the system of policy changes whose principle effect may be elsewhere.

### Limitations

There are a number of limitations to our study. Firstly, there has been a significant increase in the number of individuals identified as “mixed race” in the US [13]. That said, self-identified race/ethnicity is a strong predictor of both self-rated health and health outcomes [5, 24]. Secondly, there has been debate related to the use of ICD codes to identify health status [29]. It remains the case, however, that these are internationally recognized classifications, that are widely used [30]. Thirdly, outpatient data is not collected on the same annual basis as inpatient data by AHRQ. It was not therefore possible to examine trends in an outpatient setting or the effect on these of PPS. Data on anaemia treatment and diagnosis were not available for each admission, which limits our ability to examine the effects of these factors on recorded anaemia over time. The possibility that the protocol(s) for anaemia management and implementation may vary between transplantation candidates and other RRT groups warrants research attention. Nevertheless, the key results remained unchanged while removing the transplantation group from our main analyses (Results available upon request). Finally, the essentially cross-sectional nature of the data based on each single hospitalization also limited our ability to examine patterns related for example to readmissions.

## Conclusions

We found a significant increase in anaemia recorded as a complication among ESRD hospitalizations in the US following the adoption of PPS. We found ethnic disparities in the recorded anaemia as a complication among ESRD admissions, a gap that was most evident among Native American admissions and that widened for all racial/ethnic groups following the introduction of the PPS. The study demonstrates the potential unintended consequence of measures targeting behaviour in one part of the healthcare system to have a material impact on other parts in terms of both equity and efficiency.

## Supplementary information

**Additional file 1: Table S1.** ICD9 and ICD10 codes used for identifying study population. **Figure S1.** Temporal trends (from 2008 to 2016) in the percentage of ESRD admissions with anaemia. **Figure S2.** Predicted probability of anaemia from the fully adjusted logistic regression model across gender, insurance type, income level, and RRT types over time. **Table S2.** The results from main models without weighting variables. **Table S3.** The sensitivity analysis results from models weighted by HCUP-NIS weighting variable.

## Data Availability

The data that support the findings of this study are available from the Agency for Healthcare Research and Quality (AHRQ) but restrictions apply to the availability of these data, which were used under license for the current study, and so are not publicly available. Data are however available from the authors upon reasonable request and with permission of Agency for Healthcare Research and Quality (AHRQ). Future researchers interested in purchasing and using AHRQ-HCUP databases could visit this website: https://www.distributor.hcup-us.ahrq.gov/

## Abbreviations

AHRQ: Agency for Healthcare Research and Quality
CKD: Chronic kidney disease
ESAs: Erythropoiesis-stimulating agents
ESRD: End-stage renal disease
FDA: US Food and Drug Administration
HCUP-NIS: National (Nationwide) Inpatient Sample - Healthcare Cost and Utilization Project
ICD: International Classification of Diseases
PPS: Prospective payment system
RRT: Renal replacement therapies
