# Supplementary material for: Prospective payment system and racial/ethnic disparities: a national retrospective observational study in anaemia complication among end-stage renal disease patients in the US
Source: BMC Nephrol. 2020 Oct 6;21:423. doi: 10.1186/s12882-020-02081-4 (PMC7541203; doi:10.1186/s12882-020-02081-4)
Supplement: Supplementary file 1 — Additional file 1: Table S1. ICD9 and ICD10 codes used for identifying study population. Figure S1. Temporal trends (from 2008 to 2016) in the percentage of ESRD admissions with anaemia. Figure S2. Predicted probability of anaemia from the fully adjusted logistic regression model across gender, insurance type, income level, and RRT types over time. Table S2. The results from main models without weighting variables. Table S3. The sensitivity analysis results from models weighted by HCUP-NIS weighting variable. [file 12882_2020_2081_MOESM1_ESM.docx]

# ADDITIONAL FILE

**Variable description**

HCUP-NIS classified insurance status as Medicare, Medicaid, Private insurance, and other. Area level income provided a quartile classification of the estimated median household income of residents in the patient's ZIP Code and indicated the lowest to highest income populations. Patient location was a six-category urban-rural classification scheme for U.S. counties developed by the National Center for Health Statistic. This included "Central" counties of metro areas of >=1 million population (central counties), "Fringe" counties of metro areas of >=1 million population (large metro), Counties in metro areas of 250,000-999,999 population (medium metro), Counties in metro areas of 50,000-249,999 population (small metro), Micropolitan counties, and Not metropolitan or Micropolitan counties. In order to control for hospital characteristics, each hospital’s unique HCUP number was used to link the core data to the Hospital weights file. These variables comprised hospital's ownership/control category (government/private), location (rural/urban), and teaching status of hospital (non-teaching/teaching).

## **Table S1.** ICD9 and ICD10 codes used for identifying study population

|  | **ICD9**  **Diagnostic/Procedure codes** | **ICD10**  **Diagnostic/Procedure codes** ᶲ |
| --- | --- | --- |
| Hemodialysis | Procedure code 39.95 | Procedure codes: 5A1D00Z, 5A1D60Z |
| Peritoneal dialysis | Procedure code 54.98 | Procedure code 3E1M39Z |
| Kidney transplantation | Procedure code 55.69  Diagnostic code V42.0 | Procedure codes: 0TY00Z0, 0TY00Z1, 0TY00Z2, 0TY10Z0, 0TY10Z1, 0TY10Z2.  Diagnostic code Z94.0 |
| Anemia due to CKD | Diagnostic code 285.21 | Diagnostic code D63.1 |
| Acute kidney injury | Diagnostic codes 584.x | Diagnostic codes N17.x |
| Iron deficiency | Diagnostic code 280.9 | Diagnostic code D50.9 |

*Source: Centers for Medicare and Medicaid Services (2015), ICD-10 Procedure Coding System (ICD-10-PCS) 2015 Tables and Index,* [*https://www.cms.gov/medicare/coding/icd10/2015-icd-10-pcs-and-gems.html*](https://www.cms.gov/medicare/coding/icd10/2015-icd-10-pcs-and-gems.html)

*Note: ᶲ ICD10 Diagnostic/Procedure codes were applied for HCUP NIS data since the 4^th^ quarter of year 2015.*

## **Figure S1.** Temporal trends (from 2008 to 2016) in the percentage of ESRD admissions with anaemia


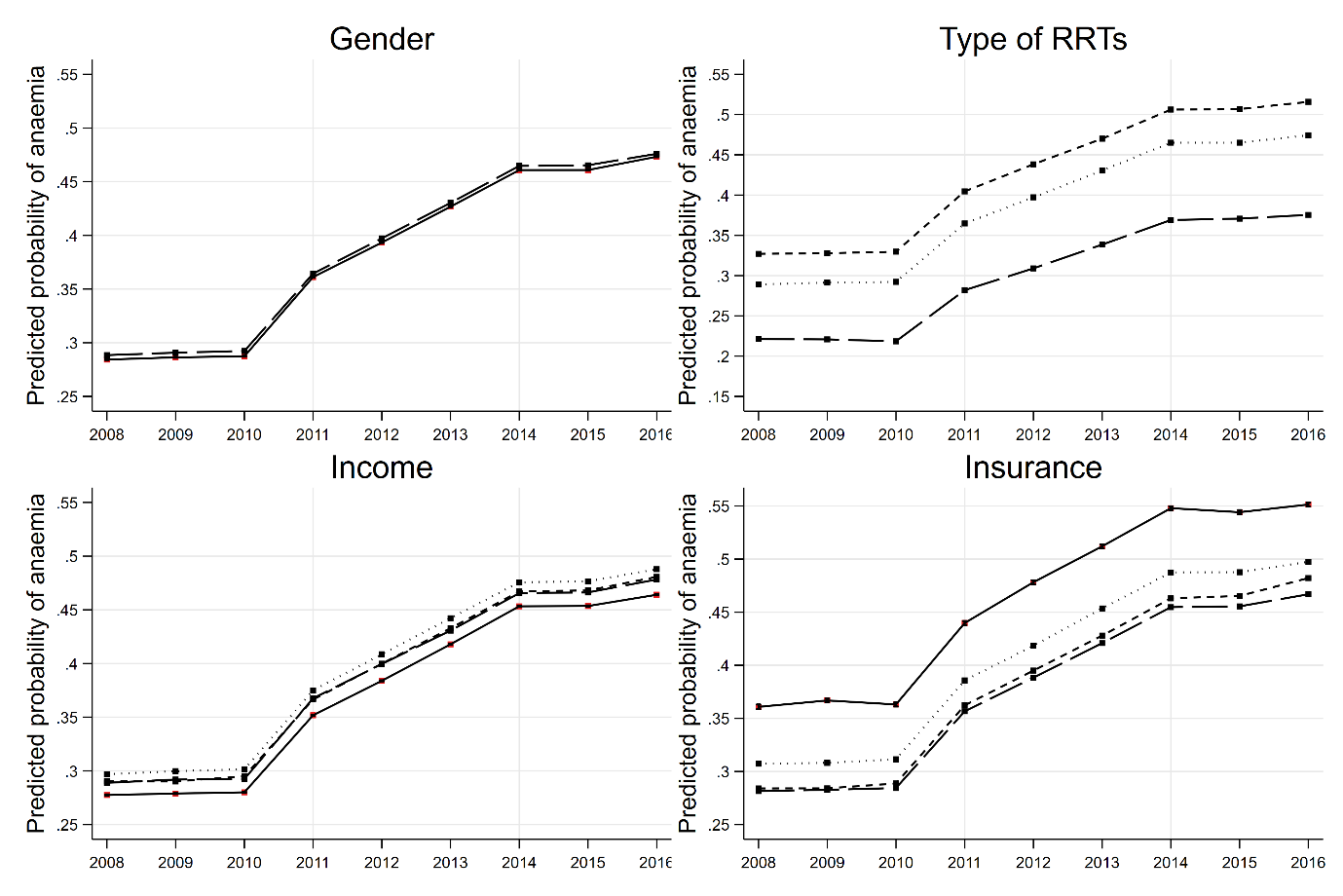


## **Figure S2.** Predicted probability of anaemia from the fully adjusted logistic regression model across gender, insurance type, income level, and RRT types over time

## **Table S2.** The results from main models without weighting variables

| **Variables** | **Model 1** | **Model 2** | **Model 3** | **Model 4** |
| --- | --- | --- | --- | --- |
| White Americans |  |  |  |  |
| Native Americans | 1.352^***^ | 1.230^***^ | 1.287^***^ | 1.196^***^ |
|  | [1.304,1.402] | [1.182,1.280] | [1.240,1.335] | [1.149,1.245] |
| Black Americans | 1.044^***^ | 1.039^***^ | 1.036^***^ | 1.040^***^ |
|  | [1.035,1.053] | [1.029,1.049] | [1.027,1.045] | [1.030,1.050] |
| Asian Americans | 1.234^***^ | 1.204^***^ | 1.210^***^ | 1.196^***^ |
|  | [1.209,1.259] | [1.179,1.230] | [1.185,1.236] | [1.171,1.222] |
| Hispanic Americans | 1.167^***^ | 1.132^***^ | 1.128^***^ | 1.115^***^ |
|  | [1.155,1.180] | [1.119,1.146] | [1.115,1.141] | [1.102,1.129] |
| Other ethnicities | 0.979 | 0.959^***^ | 0.965^**^ | 0.964^**^ |
|  | [0.957,1.002] | [0.936,0.982] | [0.943,0.988] | [0.941,0.988] |
| Other types of insurance/No insurance |  | Ref |  | Ref |
| Medicaid |  | 0.824^***^ |  | 0.817^***^ |
|  |  | [0.802,0.845] |  | [0.795,0.838] |
| Medicare |  | 0.768^***^ |  | 0.769^***^ |
|  |  | [0.750,0.787] |  | [0.751,0.788] |
| Private insurance |  | 0.790^***^ |  | 0.804^***^ |
|  |  | [0.769,0.811] |  | [0.783,0.825] |
| Female |  | 1.031^***^ |  | 1.027^***^ |
|  |  | [1.023,1.039] |  | [1.019,1.036] |
| Male |  | Ref |  | Ref |
| <=37 years old |  | Ref |  | Ref |
| 38-47 years old |  | 0.895^***^ |  | 0.875^***^ |
|  |  | [0.880,0.910] |  | [0.860,0.890] |
| 48-52 years old |  | 0.866^***^ |  | 0.830^***^ |
|  |  | [0.852,0.880] |  | [0.816,0.844] |
| 53-57 years old |  | 0.847^***^ |  | 0.809^***^ |
|  |  | [0.832,0.861] |  | [0.794,0.823] |
| 58-62 years old |  | 0.852^***^ |  | 0.805^***^ |
|  |  | [0.838,0.867] |  | [0.790,0.819] |
| 63-66 years old |  | 0.847^***^ |  | 0.799^***^ |
|  |  | [0.834,0.861] |  | [0.785,0.814] |
| 67-70 years old |  | 0.846^***^ |  | 0.792^***^ |
|  |  | [0.831,0.861] |  | [0.776,0.807] |
| 71-75 years old |  | 0.869^***^ |  | 0.810^***^ |
|  |  | [0.854,0.884] |  | [0.795,0.826] |
| 76-81 years old |  | 0.880^***^ |  | 0.821^***^ |
|  |  | [0.864,0.896] |  | [0.804,0.838] |
| >=82 years old |  | 0.898^***^ |  | 0.838^***^ |
|  |  | [0.882,0.914] |  | [0.821,0.857] |
| Income 4th quartile (highest income) |  | Ref |  | Ref |
| Income 1st quartile |  | 0.967^***^ |  | 0.961^***^ |
|  |  | [0.954,0.980] |  | [0.948,0.973] |
| Income 2nd quartile |  | 1.021^**^ |  | 1.014^*^ |
|  |  | [1.007,1.034] |  | [1.000,1.027] |
| Income 3rd quartile |  | 1.041^***^ |  | 1.035^***^ |
|  |  | [1.028,1.055] |  | [1.022,1.049] |
| Central counties of metro areas of >=1 million population |  | Ref |  | Ref |
| Fringe counties of metro areas of >=1 million population |  | 1.053^***^ |  | 1.050^***^ |
|  |  | [1.042,1.065] |  | [1.039,1.062] |
| Counties in metro areas of 250,000-999,999 population |  | 1.008 |  | 1.004 |
|  |  | [0.997,1.020] |  | [0.993,1.016] |
| Counties in metro areas of 50,000-249,999 population |  | 1.060^***^ |  | 1.052^***^ |
|  |  | [1.044,1.077] |  | [1.036,1.069] |
| Micropolitan counties |  | 0.881^***^ |  | 0.873^***^ |
|  |  | [0.867,0.895] |  | [0.858,0.889] |
| Not metropolitan or micropolitan counties |  | 1.031^**^ |  | 1.023^*^ |
|  |  | [1.012,1.051] |  | [1.003,1.044] |
| Before PPS |  | Ref | Ref | Ref |
| After PPS |  | 1.410^***^ | 1.405^***^ | 1.419^***^ |
|  |  | [1.394,1.427] | [1.388,1.422] | [1.401,1.437] |
| Iron deficiency |  |  | 1.392^***^ | 1.384^***^ |
|  |  |  | [1.355,1.430] | [1.346,1.422] |
| ACCI score |  |  | 0.994^***^ | 1.009^***^ |
|  |  |  | [0.992,0.995] | [1.007,1.012] |
| Kidney transplantation |  |  | Ref | Ref |
| Haemodialysis |  |  | 1.674^***^ | 1.686^***^ |
|  |  |  | [1.642,1.708] | [1.653,1.721] |
| Peritoneal dialysis |  |  | 1.951^***^ | 1.946^***^ |
|  |  |  | [1.900,2.003] | [1.894,1.999] |
| Private hospital |  |  | 0.960^***^ | 0.975^***^ |
|  |  |  | [0.949,0.971] | [0.964,0.987] |
| Hospital in urban area |  |  | 1.074^***^ | 1.003 |
|  |  |  | [1.059,1.090] | [0.987,1.019] |
| Teaching hospital |  |  | 0.961^***^ | 0.978^***^ |
|  |  |  | [0.952,0.969] | [0.969,0.987] |
| Proteinuria |  |  | 1.550^***^ | 1.496^***^ |
|  |  |  | [1.400,1.716] | [1.349,1.659] |
| Diabetes |  |  | 0.883^***^ | 0.893^***^ |
|  |  |  | [0.875,0.892] | [0.884,0.902] |
| Diabetes with complications |  |  | 1.201^***^ | 1.194^***^ |
|  |  |  | [1.189,1.212] | [1.182,1.206] |
| Observations | 1147786 | 1109297 | 1147786 | 1109297 |

*Note: This table presents the results from four sets of logistic regression analysis combined with linear spline in the main analysis. Model 1: no adjustment. Model 2: adjusted for demographic and socioeconomic variables Insurance type, gender, age at admission, median household income for patient's ZIP Code, location. Model 3: adjusted for clinical variables: renal replacement therapies, iron deficiency, comorbidity score ACCI, hospital characteristics, proteinuria, and diabetes with or without complications. Model 4: fully adjusted model for all demographic, socioeconomic and clinical variables. 95%CI is in the brackets. * p<0.05, ** p<0.01, *** p<0.001.*

## **Table S3.** The sensitivity analysis results from models weighted by HCUP-NIS weighting variable

| **Variables** | **Model 1** | **Model 2** | **Model 3** | **Model 4** |
| --- | --- | --- | --- | --- |
| White Americans |  |  |  |  |
| Native Americans | 1.345^***^ | 1.225^***^ | 1.281^***^ | 1.192^***^ |
|  | [1.297,1.395] | [1.177,1.275] | [1.234,1.329] | [1.145,1.241] |
| Black Americans | 1.041^***^ | 1.035^***^ | 1.033^***^ | 1.036^***^ |
|  | [1.032,1.050] | [1.025,1.045] | [1.024,1.042] | [1.026,1.047] |
| Asian Americans | 1.238^***^ | 1.206^***^ | 1.213^***^ | 1.199^***^ |
|  | [1.212,1.263] | [1.180,1.232] | [1.188,1.239] | [1.173,1.225] |
| Hispanic Americans | 1.166^***^ | 1.132^***^ | 1.128^***^ | 1.115^***^ |
|  | [1.153,1.179] | [1.118,1.146] | [1.115,1.141] | [1.101,1.129] |
| Other ethnicities | 0.988 | 0.964^**^ | 0.971^*^ | 0.970^*^ |
|  | [0.965,1.011] | [0.941,0.987] | [0.948,0.994] | [0.947,0.994] |
| Other types of insurance/No insurance |  | Ref |  | Ref |
| Medicaid |  | 0.825^***^ |  | 0.818^***^ |
|  |  | [0.804,0.847] |  | [0.796,0.840] |
| Medicare |  | 0.770^***^ |  | 0.771^***^ |
|  |  | [0.752,0.789] |  | [0.752,0.790] |
| Private insurance |  | 0.791^***^ |  | 0.805^***^ |
|  |  | [0.771,0.813] |  | [0.784,0.827] |
| Female |  | 1.031^***^ |  | 1.028^***^ |
|  |  | [1.023,1.039] |  | [1.020,1.036] |
| Male |  | Ref |  | Ref |
| <=37 years old |  | Ref |  | Ref |
| 38-47 years old |  | 0.897^***^ |  | 0.877^***^ |
|  |  | [0.882,0.913] |  | [0.862,0.892] |
| 48-52 years old |  | 0.866^***^ |  | 0.831^***^ |
|  |  | [0.852,0.880] |  | [0.817,0.845] |
| 53-57 years old |  | 0.847^***^ |  | 0.809^***^ |
|  |  | [0.832,0.861] |  | [0.795,0.824] |
| 58-62 years old |  | 0.855^***^ |  | 0.808^***^ |
|  |  | [0.840,0.869] |  | [0.793,0.822] |
| 63-66 years old |  | 0.849^***^ |  | 0.801^***^ |
|  |  | [0.835,0.863] |  | [0.787,0.816] |
| 67-70 years old |  | 0.846^***^ |  | 0.793^***^ |
|  |  | [0.831,0.862] |  | [0.777,0.809] |
| 71-75 years old |  | 0.870^***^ |  | 0.813^***^ |
|  |  | [0.855,0.885] |  | [0.797,0.829] |
| 76-81 years old |  | 0.881^***^ |  | 0.823^***^ |
|  |  | [0.865,0.897] |  | [0.806,0.840] |
| >=82 years old |  | 0.898^***^ |  | 0.839^***^ |
|  |  | [0.882,0.915] |  | [0.822,0.858] |
| Income 4th quartile (highest income) |  | Ref |  | Ref |
| Income 1st quartile |  | 0.968^***^ |  | 0.962^***^ |
|  |  | [0.955,0.980] |  | [0.949,0.974] |
| Income 2nd quartile |  | 1.023^**^ |  | 1.016^*^ |
|  |  | [1.009,1.036] |  | [1.002,1.029] |
| Income 3rd quartile |  | 1.043^***^ |  | 1.037^***^ |
|  |  | [1.029,1.057] |  | [1.023,1.051] |
| Central counties of metro areas of >=1 million population |  | Ref |  | Ref |
| Fringe counties of metro areas of >=1 million population |  | 1.052^***^ |  | 1.048^***^ |
|  |  | [1.040,1.063] |  | [1.037,1.060] |
| Counties in metro areas of 250,000-999,999 population |  | 1.005 |  | 1.001 |
|  |  | [0.993,1.016] |  | [0.989,1.012] |
| Counties in metro areas of 50,000-249,999 population |  | 1.056^***^ |  | 1.048^***^ |
|  |  | [1.040,1.073] |  | [1.031,1.064] |
| Micropolitan counties |  | 0.879^***^ |  | 0.873^***^ |
|  |  | [0.865,0.894] |  | [0.857,0.888] |
| Not metropolitan or micropolitan counties |  | 1.027^**^ |  | 1.019 |
|  |  | [1.007,1.047] |  | [0.999,1.040] |
| Before PPS |  | Ref | Ref | Ref |
| After PPS |  | 1.402^***^ | 1.394^***^ | 1.407^***^ |
|  |  | [1.385,1.419] | [1.377,1.411] | [1.389,1.425] |
| Iron deficiency |  |  | 1.386^***^ | 1.379^***^ |
|  |  |  | [1.349,1.424] | [1.341,1.417] |
| ACCI score |  |  | 0.994^***^ | 1.009^***^ |
|  |  |  | [0.992,0.995] | [1.007,1.011] |
| Kidney transplantation |  |  | Ref | Ref |
| Haemodialysis |  |  | 1.675^***^ | 1.688^***^ |
|  |  |  | [1.642,1.709] | [1.654,1.722] |
| Peritoneal dialysis |  |  | 1.953^***^ | 1.948^***^ |
|  |  |  | [1.902,2.006] | [1.896,2.002] |
| Private hospital |  |  | 0.964^***^ | 0.979^***^ |
|  |  |  | [0.953,0.975] | [0.967,0.990] |
| Hospital in urban area |  |  | 1.079^***^ | 1.008 |
|  |  |  | [1.063,1.095] | [0.991,1.024] |
| Teaching hospital |  |  | 0.957^***^ | 0.975^***^ |
|  |  |  | [0.949,0.966] | [0.966,0.984] |
| Proteinuria |  |  | 1.547^***^ | 1.493^***^ |
|  |  |  | [1.397,1.713] | [1.347,1.656] |
| Diabetes |  |  | 0.882^***^ | 0.891^***^ |
|  |  |  | [0.874,0.891] | [0.882,0.901] |
| Diabetes with complications |  |  | 1.200^***^ | 1.193^***^ |
|  |  |  | [1.189,1.212] | [1.181,1.205] |
| Observations | 1138028 | 1099774 | 1138028 | 1099774 |

Note: This table presents the results from four sets of logistic regression analysis combined with linear spline in the sensitivity analysis. Model 1: no adjustment. Model 2: adjusted for demographic and socioeconomic variables Insurance type, gender, age at admission, median household income for patient's ZIP Code, location. Model 3: adjusted for clinical variables: renal replacement therapies, iron deficiency, comorbidity score ACCI, hospital characteristics, proteinuria, and diabetes with or without complications. Model 4: fully adjusted model for all demographic, socioeconomic and clinical variables. All models were weighted with trend weight for data years prior to 2012 and the discharge-level weight for data years 2012 and later. 95%CI is in the brackets. * p<0.05, ** p<0.01, *** p<0.001.
